# Editorial

**Published:** 1839-07-06

**Authors:** 


### THE MEDICAL EXAMINER.

### PHILADELPHIA, JULY 6, 1839.

We publish some extracts from Dr. Warrington's introductory lecture on Obstetrics. The course which is pursued by that gentleman consists of practical instruction in obstetrics, together with explanatory lectures upon the subject. His classes are limited to a small number of pupils, so that each student is enabled to attend at least two cases, besides enjoying frequent opportunities of practising the various operations on the mannekin. These advantages immensely facilitate the progress of the pupil in an art where practice is all-important.

